# Multi-Purpose Utility of Circulating Plasma DNA Testing in Patients with Advanced Cancers

**DOI:** 10.1371/journal.pone.0047020

**Published:** 2012-11-07

**Authors:** Geraldine Perkins, Timothy A. Yap, Lorna Pope, Amy M. Cassidy, Juliet P. Dukes, Ruth Riisnaes, Christophe Massard, Philippe A. Cassier, Susana Miranda, Jeremy Clark, Katie A. Denholm, Khin Thway, David Gonzalez De Castro, Gerhardt Attard, L. Rhoda Molife, Stan B. Kaye, Udai Banerji, Johann S. de Bono

**Affiliations:** 1 Division of Clinical Studies, The Institute of Cancer Research, Sutton, Surrey, United Kingdom; 2 Drug Development Unit, Royal Marsden NHS Foundation Trust, Sutton, Surrey, United Kingdom; University Clinic of Navarra, Spain

## Abstract

Tumor genomic instability and selective treatment pressures result in clonal disease evolution; molecular stratification for molecularly targeted drug administration requires repeated access to tumor DNA. We hypothesized that circulating plasma DNA (cpDNA) in advanced cancer patients is largely derived from tumor, has prognostic utility, and can be utilized for multiplex tumor mutation sequencing when repeat biopsy is not feasible. We utilized the Sequenom MassArray System and OncoCarta panel for somatic mutation profiling. Matched samples, acquired from the same patient but at different time points were evaluated; these comprised formalin-fixed paraffin-embedded (FFPE) archival tumor tissue (primary and/or metastatic) and cpDNA. The feasibility, sensitivity, and specificity of this high-throughput, multiplex mutation detection approach was tested utilizing specimens acquired from 105 patients with solid tumors referred for participation in Phase I trials of molecularly targeted drugs. The median cpDNA concentration was 17 ng/ml (range: 0.5–1600); this was 3-fold higher than in healthy volunteers. Moreover, higher cpDNA concentrations associated with worse overall survival; there was an overall survival (OS) hazard ratio of 2.4 (95% CI 1.4, 4.2) for each 10-fold increase in cpDNA concentration and in multivariate analyses, cpDNA concentration, albumin, and performance status remained independent predictors of OS. These data suggest that plasma DNA in these cancer patients is largely derived from tumor. We also observed high detection concordance for critical ‘hot-spot’ mutations (*KRAS*, *BRAF*, *PIK3CA*) in matched cpDNA and archival tumor tissue, and important differences between archival tumor and cpDNA. This multiplex sequencing assay can be utilized to detect somatic mutations from plasma in advanced cancer patients, when safe repeat tumor biopsy is not feasible and genomic analysis of archival tumor is deemed insufficient. Overall, circulating nucleic acid biomarker studies have clinically important multi-purpose utility in advanced cancer patients and further studies to pursue their incorporation into the standard of care are warranted.

## Introduction

The development of cancer is primarily due to genetic aberrations that drive oncogenesis and determine the clinical manifestations of tumors; these may also impact response to treatment [1]. Our improved knowledge of the underlying biology of cancer and the availability of modern biotechnological tools is beginning to lead to the successful development of novel antitumor molecular therapeutics, as well as a better recognition of mechanisms of resistance [2], [3]. Notable examples include *KRAS* mutations in colorectal tumors predicting resistance to anti-epidermal growth factor receptor (EGFR) targeting monoclonal antibodies (cetuximab [ImClone and Bristol-Myers Squibb]; and panitumumab [Amgen]) [4], [5], and *KIT* mutations predicting antitumor responses to imatinib (Novartis) in gastrointestinal stromal tumors [6]. Molecular analysis of these genomic aberrations is usually conducted on archival tumor tissue due to ethical and safety challenges associated with repeated biopsies. However, in view of the potential for genomic instability, concerns remain, about the validity of this approach of analyzing archival tumor tissue, rather than rebiopsying tumor for molecular analyses at each therapeutic decision point. For example, it is unclear if the analysis of archival tumor biopsies taken many years and frequently multiple therapies previously, sufficiently reflects disease biology at time of treatment. Rebiopsy of a selected tumor lesion may not, however, provide sufficient information on intra-patient disease molecular heterogeneity and rebiopsying multiple lesions remains clinically impractical. Improved strategies to pursue patient molecular stratification are urgently needed.

We set out to optimize benefit for patients with advanced solid tumors referred for Phase I clinical trials by allocating specific targeted therapies to patients who harbor tumor molecular aberrations targeted by the agent in question [2], [3], [7]. We evaluated tumors obtained from these patients with the high throughput Sequenom MassArray platform utilizing the OncoCarta mutation panel (version 1.0; Sequenom, San Diego, CA). This panel utilizes pre-designed and pre-validated mass spectrometric SNP genotyping technology for the parallel multiplex analyses of 238 simple and complex mutations across 19 common oncogenes, minimizing the amount of specimen required and maximizing sensitivity [8]. It has previously been used successfully for the screening of mutations in formalin-fixed paraffin-embedded (FFPE) tumor tissue [9] [10].

An alternative source of tumor DNA is circulating plasma DNA (cpDNA) [11], which may be easily and repeatedly extracted from plasma and may be tumor-derived [11], [12], with cpDNA concentrations associating with disease burden and progression [13]. Studies have also demonstrated the feasibility of mutation detection from cpDNA in patients with advanced cancer [14], [15], [16], [17], [18]. We set out to explore the potential utility of multiplex mutation detection from cpDNA with the high throughput Sequenom MassArray platform utilizing the OncoCarta mutation panel (v1.0) to determine if this may be used as an adjunct to tissue biopsies to enrich and support tumor data for patient selection. Secondary objectives were to investigate if the measurement of cpDNA concentrations has prognostic value.

## Materials and Methods

### Clinical specimens

Patients with late stage advanced solid tumors who were referred to the Drug Development Unit in the Royal Marsden NHS Foundation Trust between September 2009 and August 2010, and who were eligible for a Phase I trial were included in this study. All patients provided written informed consent for genetic analysis of their tumors and plasma samples prior to participation in this study. Eight mls of peripheral blood were sampled in a BD Vacutainer Cell Preparation Tube (CPT) containing sodium heparin, which permits plasma and mononuclear cell separation during a single centrifugation step. The tube was inverted a minimum of 8 times to ensure thorough mixing of the sample, and then centrifuged at 1800 g for 15 min. The resultant plasma supernatant was transferred to a clean tube and stored at −80°C until analysis. In addition, 20 healthy volunteers provided 8 ml of blood for analysis using this method. Corresponding FFPE samples (primary and/or metastatic sites) for each patient were also requested. The relevant regulatory and independent ethics committee (National Research Ethics Service (NRES) Committee London-Chelsea, United Kingdom) approved this study prior to trial commencement.

### DNA isolation and quantification

For the analyses of tumor samples, hemotoxylin- and eosin-stained slides were reviewed by a board-certified pathologist (K.T.) to ensure adequate viable tumor and to determine the tumoral zone to core. DNA from FFPE specimens was extracted from 1 mm cores when possible or from 10 µm unstained sections with smaller biopsies using the QIAamp DNA FFPE Tissue Kit (Qiagen, Valencia, CA, USA), according to the manufacturer's recommendations. The extracted DNA was subsequently eluted in 30 µl of ATE buffer and stored at −20°C until further analysis. DNA was quantified using the Nanodrop 1000 Spectrophotometer (Thermo Scientific).

For cpDNA extraction, plasma was thawed at ambient temperature and cpDNA extracted from 2 ml of plasma using a QIAamp DNA Blood Midi Kit (Qiagen, Valencia, CA, USA), according to the manufacturer's instructions, with the following modifications: for each 2 ml sample of plasma, an additional centrifugation step (16000 g, 5 min, RT) was added before the extraction procedure in order to eliminate cellular debris from the plasma. At the end of the procedure, the DNA was eluted in 100 µl of AE elution buffer. DNA concentration was measured with fluorescent staining, using the Quant-iT™ Pico-Green® double stranded DNA (dsDNA) Assay Kit (Invitrogen, Carlsbad, CA) and the SynergyHT microplate reader (Biotek). DNA from the cancer cell lines analyzed was extracted from pellets using the QIAamp DNA Mini Kit (Qiagen, Valencia, CA, USA), according to the manufacturer's recommendations. For purposes of comparison, all cpDNA concentrations presented in this manuscript are expressed as ng/ml of plasma.

### Mass Spectrometry TypePLEX technology and OncoCarta panel (v1.0)

The OncoCarta panel (v1.0) consists of 24 pools of primer pairs and extension primers, and has the capacity to detect 238 mutations in 19 genes. The protocol provided by Sequenom (San Diego, CA) was followed with minor modifications. The amount of DNA added to the polymerase chain reaction (PCR) was 20 ng per reaction for FFPE DNA samples. For plasma DNA samples, 30 µl of DNA were added to 30 µl of pure water, and used for the OncoCarta panel (v1.0) processing. DNA was amplified using the OncoCarta PCR primer pools, unincorporated nucleotides were inactivated by shrimp alkaline phosphatase (SAP), and a single base extension reaction was performed using extension primers that hybridize immediately adjacent to the mutations and a custom mixture of nucleotides. Salts were removed by the addition of a cation exchange resin. Multiplexed reactions were spotted onto SpectroCHIP II arrays, and DNA fragments were resolved by MALDI-TOF on the Compact Mass Spectrometer (Sequenom, San Diego, CA).

### Data analysis

Data analysis was performed using MassArray Typer Analyzer software 4.0.4.20 (Sequenom), which facilitates visualization of data patterns and the raw spectra. Typer automates the identification of mutants by comparing ratios of the wild type peak to that of all suspected mutants and generates an OncoMutation report detailing specific mutations and the ratios of wildtype and mutation peaks. All mutations from the Oncomutation report were reviewed manually by 2 blinded operators, with selected reviewed mutations from the OncoMutation report compared and confirmed to be concordant. Manual review of mutations on all OncoCarta spectra was performed to identify “real” mutant peaks from salt peaks or other background peaks. Statistical analyses are detailed in the Supplemental Methods S1.

### FFPE mutation confirmation

*KRAS* mutations were also detected using the Therascreen KRAS mutation kit (Qiagen, Germany) based on Amplification Refractory Mutation System (ARMS)-Scorpion PCR [19]. *BRAF* V600E mutations were also detected using the Capillary electrophoresis-single strand conformation analysis (CE-SSCA). Further details are provided in the Supplemental Methods S1.

## Results

### Patient characteristics

A total of 105 patients referred for phase I trial participation were enrolled between September 2009 and August 2010 (**Table 1** **; Table S1**). One patient was subsequently found to be ineligible for Phase I trials and therefore this study as he had not exhausted all lines of available antitumor treatments. The different tumor types represented in the remaining 104 patients were colorectal cancer (CRC) (n = 25), breast cancer (n = 19), melanoma (n = 15), ovarian cancer (n = 15), castration resistant prostate cancer (CRPC) (n = 11) and other tumor types (n = 19), including non-small cell lung cancer (NSCLC), mesothelioma, sarcoma, glioblastoma, adenocarcinoma of unknown primary (ACUP), cholangiocarcinoma, and cervical, endometrial, duodenal, esophageal, pancreatic and renal cancers (**Table 1**).

**Table 1:** Patient characteristics (n = 104).*

Parameter	No. of patients* (%)
**Gender**	
Male	45 (43.3%)
Female	59 (56.7%)
**Median age, years**	56 (range 22–75)
**Tumor types**	
Colorectal cancer	25 (24.0%)
Breast cancer	19 (18.3%)
Melanoma	15 (14.4%)
Castration resistant prostate cancer	11 (10.6%)
Ovarian cancer	15 (14.4%)
Other**	19 (18.3%)
**ECOG PS at screening**	
**0**	36 (34.6%)
**1**	62 (59.6%)
**2**	6 (5.8%)

	Min	Median	Max	Mean	sd
**Follow-up time (months)**	0.3	5.8	17.5	6.1	3.7
**No. of metastatic sites**	**0**	**1**	**2**	**3**	**4+**
No. of patients	4	30	41	19	10
**Albumin**	23	34	43	34.4	4.2
**LDH**	100	202.5	3531	300.3	370.2
**cpDNA (ng/mL)** ***	0.5	17.3	1600	55.4	196.1

* One patient was subsequently found to be ineligible for this study as he had not exhausted all lines of available antitumor treatments.

** Includes non-small cell lung cancer (NSCLC), mesothelioma, sarcoma, glioblastoma, adenocarcinoma of unknown primary (ACUP), cholangiocarcinoma, and cervical, endometrial, duodenal, esophageal, pancreatic and renal cancers.

*** cpDNA was collected from 101 (97%) patients; it was not possible to draw blood from 1 patient for technical reasons and blood was not collected from 2 patients due to logistical errors.

Of the 104 patients analyzed in the study, FFPE primary tumor samples were obtained for 69 (66%) subjects, with FFPE nodal and/or metastatic tumor samples being available for a further 31 (30%) patients. cpDNA was collected from 101 (97%) patients; it was not possible to draw blood from 1 patient for technical reasons and blood was not collected from 2 patients due to logistical errors. A total of 60 patients died during follow up, while data for 44 patients were censored for purposes of this publication. The median follow up time was 5.8 months (range 0.3–17.5) (**Table 1**).

### DNA serial dilution experiments for assay development

Dilutions of DNA extracted from the *KRAS* mutant HCT116 human colon cancer cell line showed that the *KRAS* G13D mutation was reproducibly detectable by the OncoCarta v1.0 panel at DNA concentrations as low as 40 ng/ml (**Figure S1**). cpDNA was also collected from healthy volunteers (**Table 2**); in these samples, the cpDNA concentration was found to be low: median 6.5 ng/ml of plasma (range 4.5–13.3 ng/ml of plasma), and no mutations were detected in any sample. A patient with advanced breast cancer who had very high cpDNA levels (1600 ng/ml of plasma) was found to have a *PIK3CA* mutation in both FFPE and cpDNA samples; serial dilutions of this cpDNA showed that the *PIK3CA* mutation was detectable up to a concentration of 2.5 ng/ml of plasma utilizing this assay.

**Table 2:** Characteristics of healthy volunteers (n = 20).

Parameters	n (%)
**Gender**	
Male	7 (35%)
Female	13 (65%)
**Median age, years**	34 (range 25–52)

	n	min	median	max	mean	sd
**cpDNA (ng/ml)**	20	4.5	6.4	13.3	7.4	2.9

### Plasma cpDNA concentration levels and mutation detection

**Figure 1:**
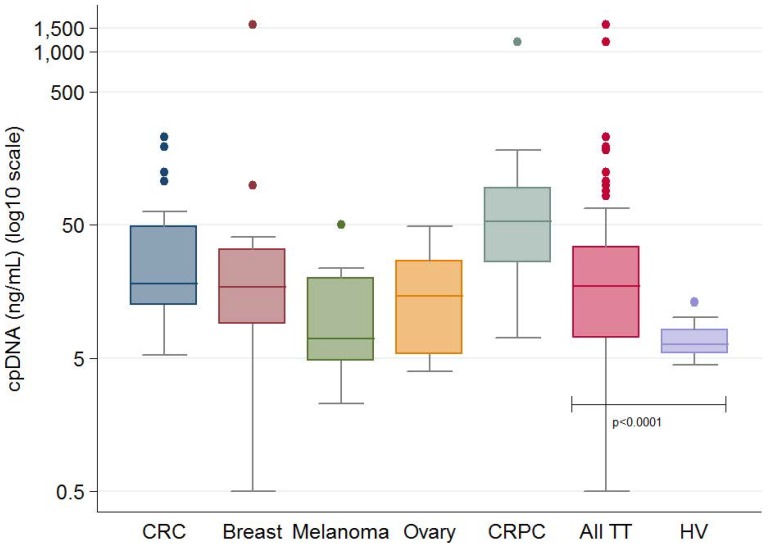
DNA concentrations (ng/mL) classified by tumor types. Box and whisker plots showing 25^th^, 50^th^ and 75^th^ percentiles, upper and lower adjacent values (whiskers) and Tukey outliers (•). P value is for a two-sided unpaired t-test on log10 DNA concentrations using Welch's correction for unequal variances.

The overall median cpDNA concentration was 17 ng/ml in these patients with advanced tumors (range: 0.5–1600) (**Figure 1** **; Table S1**). The median cpDNA concentration was 18 ng/ml (range: 5–230) for patients with CRC; 7 ng/ml (range: 2–50) for patients with melanoma, 17 ng/ml (range: 0.5–1600) for patients with breast cancer, 15 ng/ml (range: 4–49) for patients with ovarian cancer and 53 ng/ml of plasma (range: 7–1177) for patients with CRPC who had the highest plasma DNA concentrations.

**Figure 2:**
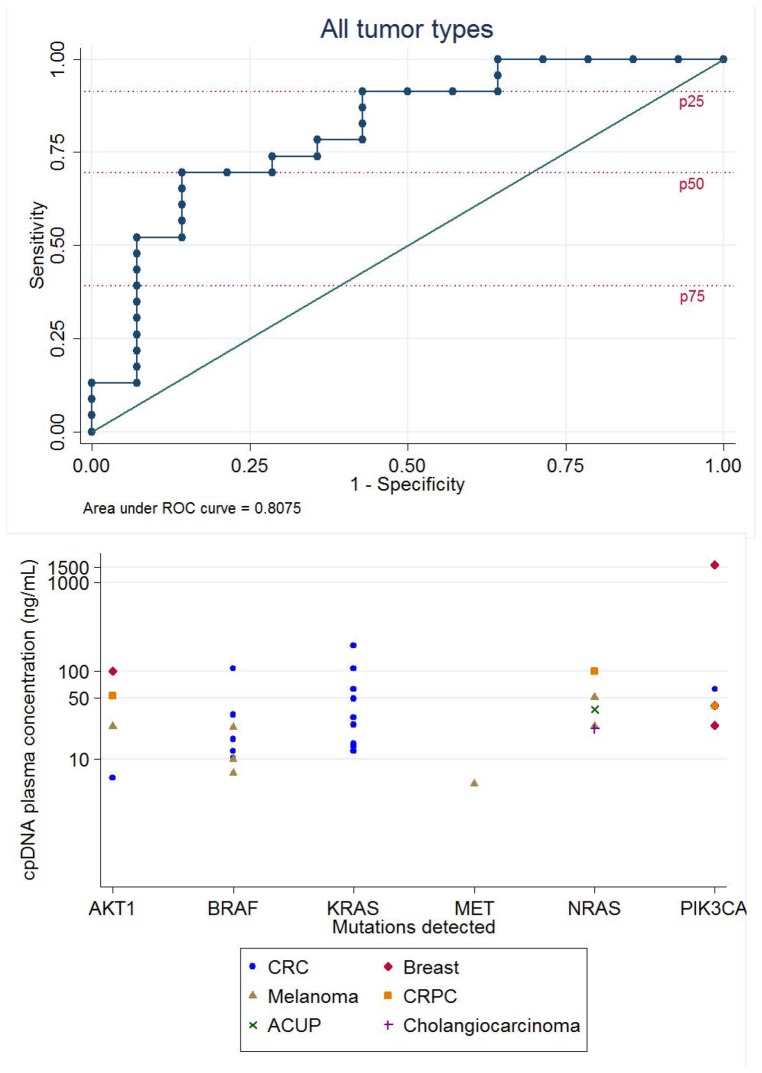
cpDNA concentrations for mutational detection by Sequenom OncoCarta panel (v1.0). **2A:** Nonparametric ROC analyses were used to assess the limit of the Sequenom platform to detect OncoCarta panel mutations in cpDNA. Each dot on the graph corresponds to the sensitivity and specificity at one of the observed concentrations. Mutations were considered ‘available for detection’ if they were detected in the patient's FFPE tissue. Mutations were detected in FFPE samples from 37 patients. The concentration of cpDNA with the optimal ability to detect a mutation is 29.95 ng/ml (Likelihood ratio = 7.3043). The AUC calculated is 0.8075 (95% CI 0.6552–0.9598). Patients whose FFPE was unavailable or tested negative for mutations were excluded from the analysis. The specificity reference lines for quartiles of DNA concentrations are indicated in red dashed lines. **2B:** Graph showing the types of mutations and cpDNA concentrations at which they were detected in different tumors. Mutations were detected in six oncogenes. Symbols represent different tumor types.

Matched plasma and FFPE were available for analysis from 84 patients. A total of 42 mutations were detected in either or both FFPE tumor and cpDNA specimens obtained from these patients (**Table 3** **; Table S1; Figures S2A–S2D**). The overall concordance in detected mutations between FFPE and cpDNA specimens was 60% (25 of 42 detected mutations) (**Table 3**). Nonparametric ROC analyses were used to assess the limit of the Sequenom platform to detect OncoCarta panel mutations in cpDNA (**Figure 2A**). The concentration of cpDNA with the optimal ability to detect a mutation was 29.95 ng/ml (Likelihood ratio = 7.3043). The AUC calculated was 0.8075 (95% CI 0.6552–0.9598). **Figure 2B** shows the different types of mutations in a range of tumor types at the respective cpDNA concentrations they were detected at.

**Table 3:** Concordance in detected mutations between paired FFPE tumors and cpDNA.

	*BRAF*	*KRAS*	*NRAS*	*HRAS*	*MET*	*AKT*	*PIK3CA*	*KIT*
**Colorectal**	3/3 (100%)	7/10 (70%)	-	-	-	1/1 (100%)	1/3 (33.3%)	-
**Melanoma**	3/5 (60%)	-	2/3 (66.7%)	-	1/1 (100%)	-	-	-
**Breast**	-	-	-	-	-	1/1 (100%)	3/4 (75%)	-
**Prostate**	-	-	0/1 (0%)	0/1 (0%)	-	1/1 (100%)	1/1 (100%)	-
**Ovarian**	-	0/2 (0%)	-	-	-	-	0/1 (0%)	0/1 (0%)
**ACUP**	-	-	1/1 (100%)	-	-	-	-	-
**Cholangiocarcinoma**	-	-	0/1 (0%)	-	-	-	-	-
**Duodenal carcinoma**	-	0/1 (0%)	-	-	-	-	-	-
**Total = 25/42 (60%)**	6/8 (75%)	7/13 (54%)	3/6 (50%)	0/1 (0%)	1/1 (100%)	3/3 (100%)	5/9 (55.6%)	0/1 (0%)

#### Correlation with patient outcome

**Figure 3:**
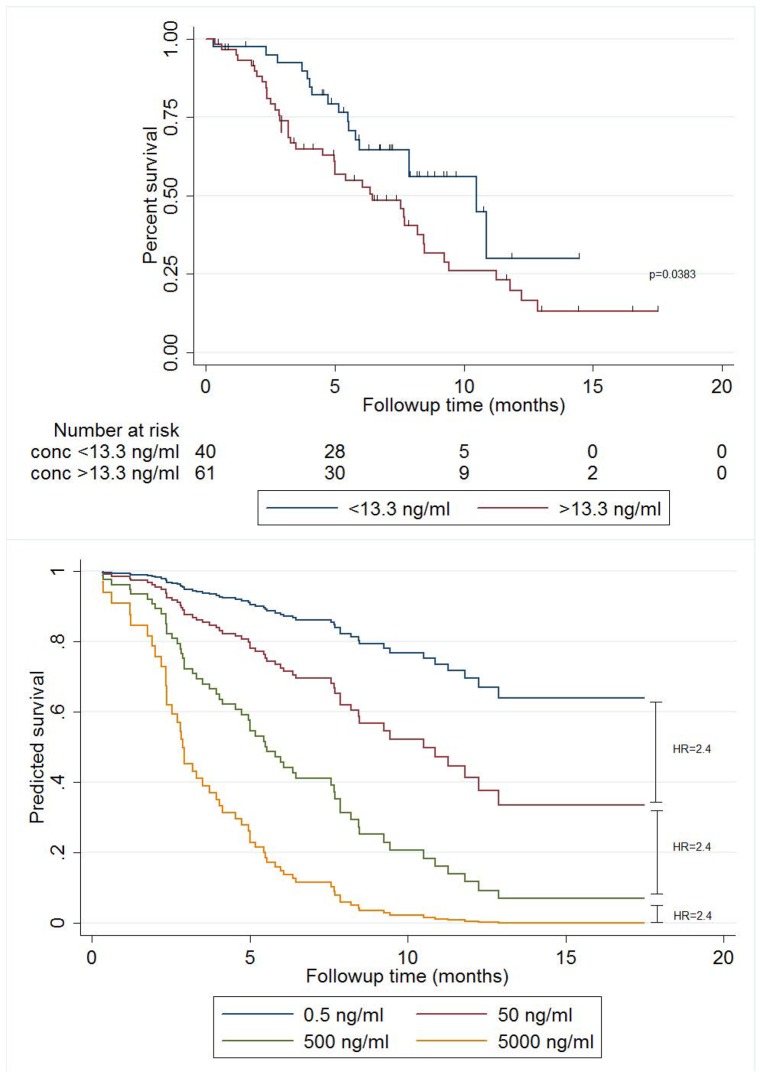
Relationship between cpDNA concentration and survival. (**3A**) Kaplan-Meier graph showing survival curves by cpDNA concentration in 101 patients with advanced solid tumors. Patients in the unfavorable category had concentrations greater than a healthy volunteer cohort maximum of 13.3 ng/ml (logrank p = 0.0383). (**3B**) Survivor function estimated from univariate Cox regression showing predicted survival curves for a range of cpDNA concentrations. A hazard ratio of 2.4 (p = 0.002) is depicted between adjacent curves.

The median overall survival (OS) for all patients was 7.9 months (95% CI 5.8, 9.2). Patients were categorised into low and high cpDNA concentration groups based on the maximum healthy volunteer cohort DNA concentration of 13.3 ng/ml; 61 patients were classified as having high cpDNA concentrations with 40 having low levels. The median OS in patients categorised as having low cpDNA concentrations was 10.5 months (95% CI 6.0, NC), while those in the high cpDNA concentration group had a median OS of 6.5 months (95% CI 4.5, 8.4) (logrank p = 0.0383) (**Figure 3A**). As a continuous variable, there was an OS hazard ratio of 2.4 (95% CI 1.4, 4.2) for each 10-fold increase in cpDNA concentration (**Figure 3B**).

#### Correlation with RMH prognostic score

**Figure 4:**
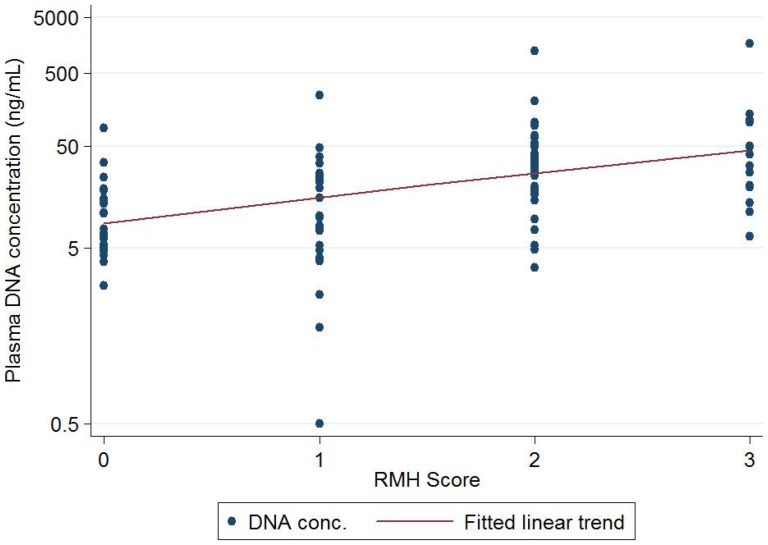
Relationship between cpDNA concentration and RMH prognostic score. Scatterplot showing the relationship between cpDNA concentration and RMH prognostic score. There was a significant positive linear trend between log_10_(cpDNA) and RMH score (beta = 0.252, p<0.0001).

We have recently prospectively validated a prognostic score (RMH score) for patients participating in Phase I clinical trials based on the combination of three prognostic factors: serum albumin less than 35 g/L; lactate dehydrogenase (LDH) greater than the upper limit of normal (ULN); and two or more sites of metastases. The presence of each of these variables associated with worsening outcome [20]. The mean cpDNA concentration was higher in patients with a worse RMH prognostic score (F[3,98] = 9.97, p<0.0001); Post-tests revealed a significant positive linear trend between log10(cpDNA) and RMH score (beta = 0.247, p<0.0001) (**Figure 4**).

#### Correlation with univariate and multivariate analysis

Univariate testing was used to determine significant predictors of overall survival, which included cpDNA concentration as a continuous variable (HR 2.4 per 10-fold increase, 95% CI 1.4–4.2), albumin <35 g/L (logrank p = 0.0003), and ECOG performance status equal to 2 (logrank p = 0.0007). When cpDNA, albumin and performance status were incorporated into a multivariate model, all three parameters were found to be independent predictors of survival (**Table 4**). The number of metastatic sites was not found to be a significant predictor of survival in the univariate analysis and was therefore excluded from the multivariate model.

**Table 4:** Univariate and multivariate analysis.

	Univariate logrank	Multivariate Cox regressionn = 101		
Variable	p	HR	95% CI	p
**cpDNA ng/mL**	(Cox regression) 2.43 (1.39–4.25) p = 0.002	1.98	1.01–3.88	0.045
**albumin <35 g/L**	0.0003	1.86	1.01–3.42	0.047
**ECOG PS = 2**	0.0007	8.05	2.53–25.65	<0.0005

### Mutational detection and concordance between FFPE and cpDNA

#### Colorectal cancer

Of 25 patients with CRC, cpDNA samples were obtained from all patients, while FFPE tumor samples were available for analysis for 22 patients. Overall, mutations were detected in 15 of 22 (68.2%) available FFPE tumors and 14 of 25 (56%) cpDNA specimens (**Table S1**). Specifically, *KRAS*, *BRAF* and *PIK3CA* mutations were detected in 10 (45%), 3 (14%) and 2 (9%) tumor specimens, respectively. Comparatively, 9 (36%) *KRAS*, 3 (12%) *BRAF* and 3 (12%) *PIK3CA* mutations were detected in cpDNA samples.

Concordance in the detection of mutations between matched FFPE archival tumors and cpDNA specimens by Sequenom OncoCarta analyses was 70% (7 of 10 patients) for *KRAS* and 100% (3 of 3 patients) for *BRAF* mutational status (**Table 3**). No patients with wildtype *KRAS* or *BRAF* tumor tissue genotypes had mutations in their respective cpDNA. Five patients had detectable *PIK3CA* mutations in either or both FFPE tumor and/or cpDNA: 1 patient had a Q546K mutation detected in both FFPE tissue and cpDNA; 1 patient had an E545K mutation detected only in FFPE, but not cpDNA; 1 patient had an E542K mutation detected in a liver metastasis (FFPE), but not in the primary tumor (FFPE) or cpDNA; 1 patient had E545K detected only in plasma but not FFPE; and 1 patient had a Q546K mutation found in cpDNA but no FFPE specimen was available. The recently reported oncogenic *AKT1* E17K mutation [21] was detected in 1 patient in both tissue and plasma. No mutations in other tested oncogenes were detected.

There was 90% (9 of 10 *KRAS* mutated samples) concordance for FFPE tumoral *KRAS* mutational status between the OncoCarta panel and the ARMS-Scorpion PCR platforms. The BRAF concordance between the OncoCarta panel and CE-SSCA method was 100% (3 of 3 *BRAF* mutated samples).

#### Melanoma

Of the 15 patients with melanoma, FFPE tumor samples were available for analysis for 10 patients, while cpDNA samples were obtained from all 15 patients. Overall, mutations were detected in 8 of 10 (80.0%) available FFPE tumors and 6 of 15 (40%) cpDNA specimens (**Table S1**).

*BRAF*, *NRAS* and *MET* mutations were detected in 5 (50%), 3 (30%) and 1 (10%) of 10 FFPE tumor specimens, respectively, and 3 (20%), 2 (13.3%) and 2 (13.3%) of 15 cpDNA samples, respectively. Concordance in the detection of mutations between matched FFPE and cpDNA was 60% (3 of 5 patients) for *BRAF*, 66.7% for *NRAS* (2 of 3 patients) and 100% for *MET* mutational status (1 of 1 patient) (**Table 3**). Another *MET* mutation, T992I, was found in one cpDNA sample, but no FFPE tumor specimen was available. No patients with wildtype tumor tissue genotypes had mutations in their respective cpDNA.

There was 100% concordance (5 of 5 samples) for the *BRAF* mutational status between the OncoCarta panel and CE-SSCA method.

#### Breast cancer

FFPE tumor samples and cpDNA samples were available for analysis for all 19 patients with breast cancer. Overall, mutations were detected in 5 of 19 (26.3%) FFPE tumors and 4 of 19 (21.1%) cpDNA specimens (**Table S1**).

The *PIK3CA* H1047R mutation was detected in 4 of 19 (21.5%) tumor specimens and 3 of 19 (15.8%) cpDNA samples, with concordance between 3 of 4 (75%) matched FFPE and cpDNA specimens (**Table 3**). The *AKT1* E17K mutation was detected in 1 patient in both FFPE tissue and cpDNA. No mutations in any of the other oncogenes studied were detected with the OncoCarta panel. No patients with wildtype tumor tissue genotypes had mutations in their respective cpDNA.

#### Castration resistant prostate cancer

Of the 11 patients with CRPC, cpDNA samples were obtained from all patients, while FFPE tumors were available for 8 patients. Overall, mutations were detected in 3 of 8 (37.5%) FFPE tumors, and 3 of 11 (27.3%) cpDNA specimens (**Table S1**).

*PIK3CA*, *HRAS* and *AKT1* (all n = 1) mutations were detected in FFPE tumor specimens, while *NRAS*, *PIK3CA* and *AKT1* (all n = 1) mutations were found in cpDNA samples. The corresponding FFPE tumor *PIK3CA* and *AKT1* mutations were found in the cpDNA samples, but the FFPE tumor *HRAS* mutation was not found in the matched cpDNA sample (**Table 3**). The Q61K *NRAS* mutation was found in 1 cpDNA specimen, but not in the corresponding FFPE tumor sample.

#### Ovarian cancer

Of the 15 patients with advanced ovarian cancer, cpDNA samples were obtained from all patients, while FFPE tumor samples were available for 14 patients. Overall, mutations were detected in 5 of 14 (35.7%) FFPE tumors, and 0 of 14 (0%) cpDNA specimens (**Table S1**).

*KRAS* mutations (G12V and G13D) (n = 3) and the *PIK3CA* H1047R mutation (n = 1) were detected in FFPE tumor samples, but no mutations were found in any cpDNA samples (**Table 3**). One patient with ovarian carcinosarcoma had a *KIT* P585P mutation detected in FFPE, but not in cpDNA.

#### Other tumor types

Of the remaining 19 patients with a range of tumor types, cpDNA samples were obtained from 17 patients, while FFPE tumors were available for 12 patients.

The *NRAS* G12D mutation was found in both FFPE tumor and plasma from a patient with ACUP (**Table 3**). The *NRAS* G13R mutation was detected in the plasma, but not in FFPE tumor from a patient with cholangiocarcinoma. The *KRAS* G12D mutation was found in FFPE tumor, but not in plasma from a patient with duodenal cancer. No mutations were detected in the other patients, including no epidermal growth factor receptor (*EGFR*) mutations in the 5 patients with NSCLC.

### Concordance in mutation detection between FPFE and cpDNA for primary tumor or metastatic specimens

When considering all patients with matched samples, including those with no mutations detected (n = 83), the concordance in detecting mutations between FFPE and cpDNA was higher in metastases (83.3% of 18 specimens) compared with primary tumor (78.5% of 65 specimens). When considering only patients with mutations detected in at least blood and/or primary tumor (n = 40), the concordance in detecting mutations between FFPE and cpDNA was again higher in metastases (70.0% of 10 specimens) compared with primary tumor (53.3% of 30 specimens). However, because of the difference in the number of primary tumor (n = 65) and metastatic (n = 18) specimens obtained, we are unable to draw any statistical conclusions from these data.

## Discussion

This study has demonstrated, for the first time, the feasibility of multiplex detection of tumor DNA mutations utilizing the multiplex OncoCarta panel from both DNA extracted from FFPE archival tumor tissue and cpDNA. We have shown that total cpDNA levels in patients with advanced cancers are, in general, significantly higher than those in healthy volunteers, with the highest concentrations found in patients with advanced prostate and breast cancers, although this difference was not significant in melanoma and ovarian cancer (**Figure 1**). The maximum concentration detected in healthy volunteers was found to split patients into two groups that were associated with significantly different prognoses; patients in the low cpDNA group had a significantly higher OS relative to those in the high cpDNA group [11], [13], [22]. Furthermore, the cpDNA concentration remained highly prognostic for OS in a multivariate analyses utilizing the prognostic biomarkers for the Phase I trial patient population that we have previously described [20]. We have also shown a correlation between cpDNA concentrations and the prognostic score that we have previously described to predict the outcome of patients referred for Phase I trial participation; cpDNA concentration, albumin <35 g/L and performance status had prognostic value in our series of patients as independent predictors of survival. These data overall indicate that cpDNA in this patient population is largely tumor derived, although this may be generated by both malignant and stromal cells.

The Sequenom OncoCarta panel has also enabled us to analyze more than 230 known mutation ‘hot-spots’ mutations in over a hundred patients in a high throughput fashion. The OncoCarta panel covers a large and increasing number of oncogenes and can be adapted to include additional genes of interest. It allows tumor mutation detection even with minimal amounts of tumor DNA, poor tissue preservation and the presence of significant amounts of normal DNA. Next generation sequencing technology will allow more DNA coverage and data acquisition, allowing the sequencing of hundreds of full length genes, which will be critical to the study of genes where mutations can be found in multiple disparate locations, as is the case for many tumor suppressor genes such as BRCA1, BRCA2, p53 and PTEN.

As we move towards the development of molecularly targeted agents for selected populations of patients, it is crucial that the molecular characterization of tumors for the prediction of efficacy to targeted therapies is incorporated into early clinical trials [2], [3]. Such an approach can increase the odds of individual patient benefit, decreasing the number of patients receiving ineffective therapies and expediting the clinical qualification of predictive biomarkers. Archival tumor tissue, frequently taken many years before, is often used for these analyses. Tumor rebiopsy remains uncommon, although it is feasible as demonstrated in the first BATTLE lung cancer adaptive trial [23]. Nevertheless, mandating multiple repeated tumor rebiopsies poses logistical, fiscal and ethical issues, while slowing down trial accrual and not addressing the issue of intra-patient lesion-to-lesion heterogeneity. Rebiopsy and cpDNA analyses can both address concerns surrounding tumor genomic instability and clonal molecular evolution due to therapeutic selection pressures while also potentially interrogating the intra-patient heterogeneity issue. Testing cpDNA has multiple advantages, being inexpensive, relatively simple to test, more acceptable to the patient and easily analyzed repeatedly permitting the study of mechanisms of drug resistance at tumor progression. Our data now support a concerted effort to develop cpDNA multi-purpose biomarker studies that can be incorporated into the standard of care.

The origin of DNA in the plasma of patients with cancer remains undefined, but may be derived from tumor cell fragments, microparticles or exosomes, or indeed from circulating free DNA [11], [22], [24]. Our studies show that the sensitivity of this technology to detect tumor mutations in cpDNA depended, in part, on cpDNA concentrations. Work carried out by other investigators through cell mixing experiments have shown that for *KRAS* mutations G12C, G1A, and G13C, the sensitivity of the Sequenom Oncocarta assay is 2.5% and for G13D it is 10% of mutant DNA [25].

The discordance in results observed between archival tumors and cpDNA specimens could be due to several reasons: 1) Poor mutation detection in cpDNA due to low cpDNA concentrations; 2) Poor DNA quality in the FFPE archival sample; 3) potential false positive results in either sample type; 4) true disease heterogeneity. Overall, however, there was high concordance rate for FFPE *KRAS* mutation detection rate between the Sequenom OncoCarta platform and the ARMS Scorpion and CE-SSCA methods suggesting that poor DNA quality in the FFPE sample was not a major issue. In addition, the mutational concordance rates between FFPE tumor samples and cpDNA observed in our study are comparable to other published methods. For example, in patients with NSCLC, the PCR-RFLP method could detect *KRAS* mutations in plasma with a concordance rate between FFPE tumors and cpDNA of 76.7% [26]. In patients with CRC, the BEAMing method has been demonstrated to detect *KRAS* mutation in plasma in 50% of patients with mutation detected in FFPE [27]. In patients with melanoma, the BEAMing platform has also been shown to detect *BRAF* mutations with a 75% concordance rate, although in the reported study, data were not available for up to a third of the tumor samples [14]. Overall, nevertheless, it is important to note that inter-assay (FFPE versus plasma) discordance may potentially be due to true tumor heterogeneity. Low cpDNA concentrations can however limit the detection of mutations in plasma, but this challenge may potentially be resolved by utilizing higher starting plasma volumes. It is important to note that in this study, several patients had cpDNA levels below that of healthy volunteers, so any tumor-derived circulating DNA in these patients is likely to be heavily diluted by genomic sequence from normal or stromal tissues.

In conclusion, we envision that biomarker studies such as that described above can have a potential impact on molecular stratification and patient care. We therefore recommend a concerted effort by the cancer community in order to develop analytically validated assays on cpDNA that can be clinically qualified for more broad utilization [2], [3]. We envision that the analyses of cpDNA will become part of cancer patient standard of care. Finally, with whole exome and cancer genome analyses becoming increasingly feasible and affordable, studies to analyze the feasibility of a deep sequencing approach from the plasma of cancer patients are now warranted [28].

## Supporting Information

Table S1 Complete data set including list of FFPE tumor and cpDNA mutations, cpDNA concentrations and tumor characteristics.
(XLS)

Figure S1 **KRAS G13D mutation peak in DNA from HCT116 cell line at 0.04 ng/µl dilution in water.** Several tumor cell lines were used to assess the performance of the OncoCarta panel (v1.0). In order to determine the sensitivity of the technique, DNA extracted from the HCT116 human colon cancer cell line was processed at a range of dilutions (with water) from 10 ng/µl to 0.01 ng/µl for *KRAS* G13D and *PIK3CA* H1047R mutations. This spectrum shows a KRAS G13D mutation peak detected in DNA from HCT116 cell line at 0.04 ng/µl dilution in water.
(JPEG)

Figure S2 **Examples of spectra of mutant peaks detected in plasma cpDNA.** **2A:** *KRAS* G13D mutation in patient with colorectal cancer; **2B:** *BRAF* V600E mutation in patient with melanoma; **2C:** *PIK3CA* mutations (H1047R and H1047L) in patient with breast cancer; **2D:** *AKT1* E17K mutation in patient with colorectal cancer.
(TIFF)

Supplemental Methods S1 **Cell lines used to assess the performance of the OncoCarta panel (v1.0), FFPE mutation confirmation, and statistical analysis.**
(DOC)
