# Supplementary figures and images for: Multi-Purpose Utility of Circulating Plasma DNA Testing in Patients with Advanced Cancers
Source: PLoS One. 2012 Nov 7;7(11):e47020. doi: 10.1371/journal.pone.0047020 (PMC3492590; doi:10.1371/journal.pone.0047020)

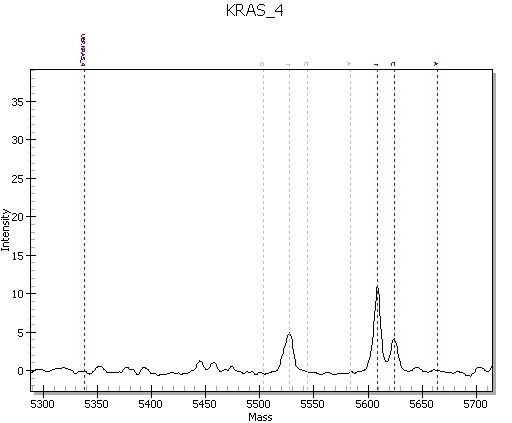

Supplement: Figure S1 — KRAS G13D mutation peak in DNA from HCT116 cell line at 0.04 ng/µl dilution in water. Several tumor cell lines were used to assess the performance of the OncoCarta panel (v1.0). In order to determine the sensitivity of the technique, DNA extracted from the HCT116 human colon cancer cell line was processed at a range of dilutions (with water) from 10 ng/µl to 0.01 ng/µl for KRAS G13D and PIK3CA H1047R mutations. This spectrum shows a KRAS G13D mutation peak detected in DNA from HCT116 cell line at 0.04 ng/µl dilution in water. (JPEG) [file pone.0047020.s002.jpeg]

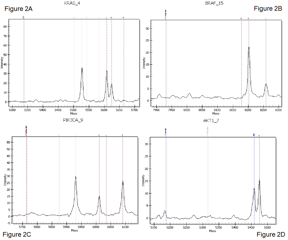

Supplement: Figure S2 — Examples of spectra of mutant peaks detected in plasma cpDNA. 2A: KRAS G13D mutation in patient with colorectal cancer; 2B: BRAF V600E mutation in patient with melanoma; 2C: PIK3CA mutations (H1047R and H1047L) in patient with breast cancer; 2D: AKT1 E17K mutation in patient with colorectal cancer. (TIFF) [file pone.0047020.s003.tiff]
