# Supplementary material for: Multi-Purpose Utility of Circulating Plasma DNA Testing in Patients with Advanced Cancers
Source: PLoS One. 2012 Nov 7;7(11):e47020. doi: 10.1371/journal.pone.0047020 (PMC3492590; doi:10.1371/journal.pone.0047020)
Supplement: Supplemental Methods S1 — Cell lines used to assess the performance of the OncoCarta panel (v1.0), FFPE mutation confirmation, and statistical analysis. (DOC) [file pone.0047020.s004.doc]

**SUPPLEMENTAL METHODS**

**Cell lines**

Several tumor cell lines were used to assess the performance of the OncoCarta panel (v1.0). All human cancer cell lines were purchased from the American Type Culture Collection apart from the renal cell carcinoma cell line SN12C [28] (kindly provided by Dr George Thomas, Portland, OR) and grown in their recommended culture medium, containing 10% fetal bovine serum (FBS) at 37°C in an atmosphere of 5% CO_2_ and passaged for less than six months. In order to determine the limit of detection of the assay, DNA extracted from the HCT116 human colon cancer cell line was processed at a range of dilutions (with water) from 10ng/μl to 0.01ng/μl for *KRAS* G13D and *PIK3CA* H1047R mutations. DNA from a mixture of 5 cell lines (SN12C, HCT116, MCF-7 human breast cancer, and the human melanoma cells SK-MEL-28 and SK-MEL-2) with known genetic mutations was used as a positive control.

**FFPE mutation confirmation**

*KRAS* mutations were also detected using the Therascreen KRAS mutation kit (Qiagen, Germany) based on Amplification Refractory Mutation System (ARMS)-Scorpion PCR [19]. *BRAF* V600E mutations were also detected using the Capillary electrophoresis-single strand conformation analysis (CE-SSCA). Amplification of exon 15 of *BRAF* was performed using 10ng to 100ng of DNA in a total volume of 25µL containing 200nmol/L of primers, 3mmol/L of MgCl_2_, 200 µmol/L of dNTPs (Invitrogen) and 0.5 units of Platinum Taq DNA Polymerase (Invitrogen). Primers sequences were adapted from benlloch et al [29]: BRAF-51-F 5’ CTA CTG TTT TCC TTT ACT TAC TAC ACC TCA GA 3’ and BRAF-176-Rv ATC CAG ACA ACT GTT CAA ACT GAT G 3’. Both primers were labeled with HEX for visualization on the 3130xL sequencer (Applied Biosystems). PCR conditions were 94°C for 5 minutes followed by 35 cycles at 94°C for 30 seconds, 50°C for 30 seconds, 72°C for 30 seconds and a final extension at 72°C for 5 minutes. After amplification, 1µL of the PCR product was mixed with formamide and LIZ600 size standard (Applied Biosystems), denatured and run on a 3130xL genetic analyser with non-denaturing polymer containing 5% CAP (Applied Biosystems), 1x running buffer and 10% glycerol. In order to detect all possible mutations, the products were run at 3 different temperatures (18°C, 20°C and 25°C). Any *BRAF* mutations, including p.V600E, were detected using this method by a specific change in mobility of the mutant product at each temperature compared to the wild type.

**Statistical Analysis**

Survival experiences were estimated using the Kaplan-Meier method with logrank tests and with uni- and multivariate Cox regression. One way Analysis of Variance (ANOVA) was used for multiple means testing. Nonparametric ROC analyses were used to assess the limit of our ability to detect OncoCarta panel (v1.0) mutations in cpDNA by the concentration circulating in plasma. Likelihood ratios for mutational detection were calculated at each cutpoint: the probability of declaring the concentration sufficient to detect mutations among those with a detected mutation divided by the probability of declaring the concentration sufficient for mutational detection among those without a detected mutation. Mutations were considered ‘available for detection’ if they were detected in the patient’s FFPE tissue. Patients whose FFPE was unavailable or tested negative for mutations were excluded from the analysis. cpDNA concentration was log_10_ transformed in all parametric analyses.
